# Update on Neutrophil Function in Severe Inflammation

**DOI:** 10.3389/fimmu.2018.02171

**Published:** 2018-10-02

**Authors:** Esmaeil Mortaz, Shamila D. Alipoor, Ian M. Adcock, Sharon Mumby, Leo Koenderman

**Affiliations:** ^1^Department of Immunology, Faculty of Medicine, Shahid Beheshti University of Medical Sciences, Tehran, Iran; ^2^Clinical Tuberculosis and Epidemiology Research Center, National Research Institute of Tuberculosis and Lung Diseases, Shahid Beheshti University of Medical Sciences, Tehran, Iran; ^3^Molecular Medicine Department, Institute of Medical Biotechnology, National Institute of Genetic Engineering and Biotechnology, Tehran, Iran; ^4^Priority Research Centre for Asthma and Respiratory Disease, Hunter Medical Research Institute, University of Newcastle, Newcastle, NSW, Australia; ^5^Airways Disease Section, Faculty of Medicine, National Heart and Lung Institute, Imperial College London, London, United Kingdom; ^6^Laboratory of Translational Immunology, Department of Respiratory Medicine, University Medical Centre Utrecht, Utrecht, Netherlands

**Keywords:** neutrophils, infection, CD64, innate immunity, severe inflammation, trauma

## Abstract

Neutrophils are main players in the effector phase of the host defense against micro-organisms and have a major role in the innate immune response. Neutrophils show phenotypic heterogeneity and functional flexibility, which highlight their importance in regulation of immune function. However, neutrophils can play a dual role and besides their antimicrobial function, deregulation of neutrophils and their hyperactivity can lead to tissue damage in severe inflammation or trauma. Neutrophils also have an important role in the modulation of the immune system in response to severe injury and trauma. In this review we will provide an overview of the current understanding of neutrophil subpopulations and their function during and post-infection and discuss the possible mechanisms of immune modulation by neutrophils in severe inflammation.

## Introduction

Neutrophils are polymorphonuclear and phagocytic leukocytes that comprise the first line of host immune response against invading pathogens (1). They are also important effector cells during tissue injury-induced inflammation (2). Neutrophils have a high potency and efficacy to sense and eradicate microbial infections, and individuals with a neutrophil deficiency (such as neutropenia) are more susceptible to microbial and fungal infections (3).

Infections and their associated inflammatory mechanisms are accompanied by a rapid influx of neutrophils from the peripheral blood to the inflammatory site. There they engage and kill microorganisms and clear infections via a number of different mechanisms including chemotaxis, phagocytosis, release of reactive oxygen species (ROS), and granular proteins and the production and liberation of cytokines (4, 5). In addition to these well-established mechanisms, several reports have demonstrated the importance of neutrophil extracellular traps (NETs) in this process.

In addition to the pivotal role of neutrophils in innate immunity a large body of evidence has indicated the importance of neutrophils in the modulation of the adaptive immune response (6). Neutrophils are involved in immune regulation during both the innate and adaptive immune responses and are, therefore, considered as therapeutic targets in several diseases such as atherosclerosis (7, 8).

Although neutrophils have long been considered as a homogenous population with a conserved phenotype and function, recent evidence has demonstrated the presence of neutrophil heterogeneity with the identification of different functional phenotypes especially in cancer and inflammation (3, 9–11). Neutrophils show a spectrum of phenotypes and/or functional states. These are characterized by the expression of a wide range of cell-surface receptors that determine their function. These phenotypes seem to rapidly adapt to changes in environmental signals or triggers (12) and the expression profiles of neutrophil receptors can reflect the type and severity of the inflammatory response after severe injury (12). Since neutrophils are the main effector cells during the systemic inflammatory response (SIRS) to severe injury, neutrophil sub-phenotyping may provide both insight into disease mechanisms and be a useful risk assessment tool (12).

Although several neutrophil phenotypes exist with specialized functions, phenotypically homogenous populations with functional heterogeneity can be found in health and disease (10). However, it is unclear whether these cells originate from distinct bone marrow lineages or have undergone local differentiation (12, 13). These emerging properties of neutrophils provide us with new insight for further understanding of their roles in homeostasis and disease. We review here the roles and function of neutrophils in modulating the immune response during inflammation and summarize the mechanisms behind these processes.

## Definition, properties, and life cycle of neutrophils

Neutrophils are the most abundant circulating leukocyte population in the human immune system contributing about 50–70% of all circulating leukocytes in healthy adults (14). Neutrophils not only kill microorganisms through phagocytosis, degranulation, and the generation of NETs, but they also modulate the immune response by interacting with other immune cells such as lymphocytes and antigen presenting cells (APC) (6, 15). In addition, recent studies indicate that neutrophils show plasticity characterized by e.g., transdifferentiation to neutrophil-dendritic cell hybrids (16).

### Neutrophil life cycle

**Figure 1:**
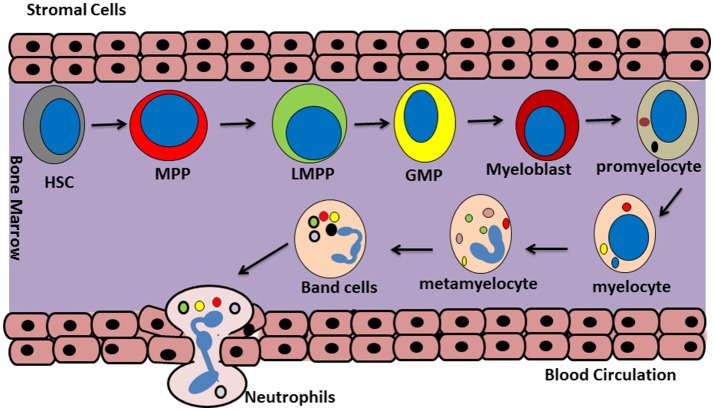
Neutrophils generation. Granulopoiesis or neutrophil generation occur in the bone marrow. At the first step, a self-renewing hematopoietic stem cell (HSC) differentiate to a multipotent progenitor (MPP) cell. Then MPP differentiate to lymphoid-primed multipotent progenitors (LPMP), which give rise into granulocyte-monocyte progenitors (GMP). After that, GMP cells turn in to myeloblast and posses through a maturation process including promyelocyte, myelocyte, metamyelocyte, band cell, and finally will commit to generate the mature neutrophils.

Neutrophil generation from committed hemopoietic progenitor cells in the bone marrow is a highly controlled process that is regulated by different transcriptional factors such as C/EBP (17, 18). At the start of this process, a self-renewing hematopoietic stem cell (HSC) differentiates into a multipotent progenitor cell (MPP) which then, in turn, transforms into lymphoid-primed multipotent progenitor cells (LMPPs). LMPPs can finally give rise to granulocyte–monocyte progenitors (GMPs) (17). GMPs undergo neutrophil generation under the influence of various growth factors such as granulocyte colony-stimulating factor (G-CSF). This occurs in a step-wise process involving progression through promyelocyte, myelocyte, metamyelocyte, and finally band neutrophil stages during which developing neutrophils gradually acquire their mature phenotype [(16); Figure 1]. During these steps, it is thought that the expression of integrin α4β1 (VLA4) and CXCR4 (at least in mice) is downregulated and that expression of CXCR2 and Toll-like receptor 4 (TLR4) is increased. During this maturation neutrophils also attain their nuclear lobular morphology (19). The formation of granules inside the developing neutrophils starts between the myeloblast and promyelocyte stage and different granules are formed at different steps of the maturation process (20). A large pool of mature neutrophils is present in bone morrow from where they can be rapidly released into the circulation in response to infectious, inflammatory or tissue damage associated stimuli (21).

The number of neutrophils in the circulating blood is regulated by the CXCL12/CXCR4 axis in the mouse (22). Under normal conditions it is estimated that approximately 10^11^ mature neutrophils leave the bone marrow and enter the circulation each day (17, 21). Bone marrow stromal cells express the chemokine CXCL12, a ligand for CXCR4 which is thought to be expressed on bone marrow neutrophils and keeps them within the bone marrow (23). Although direct evidence of CXCR4 expression on human neutrophils in the bone marrow is lacking, the CXCR4 receptor antagonist plerixafor results in the mobilization of neutrophils into the blood (23, 24). Disruption of the CXCR4/CXCL12 balance such as that found in WHIM syndrome (Warts, Hypogammaglobulinemia, Immunodeficiency, and Myelokathexis syndrome) leads to deregulated neutrophil release into the circulation (24, 25). CXCR2 signaling can act as a functional CXCR4 antagonist to control neutrophil egress from the bone marrow into blood in mice (24, 25). This needs to be confirmed in man.

### Neutrophil access to inflammatory sites

**Figure 2:**
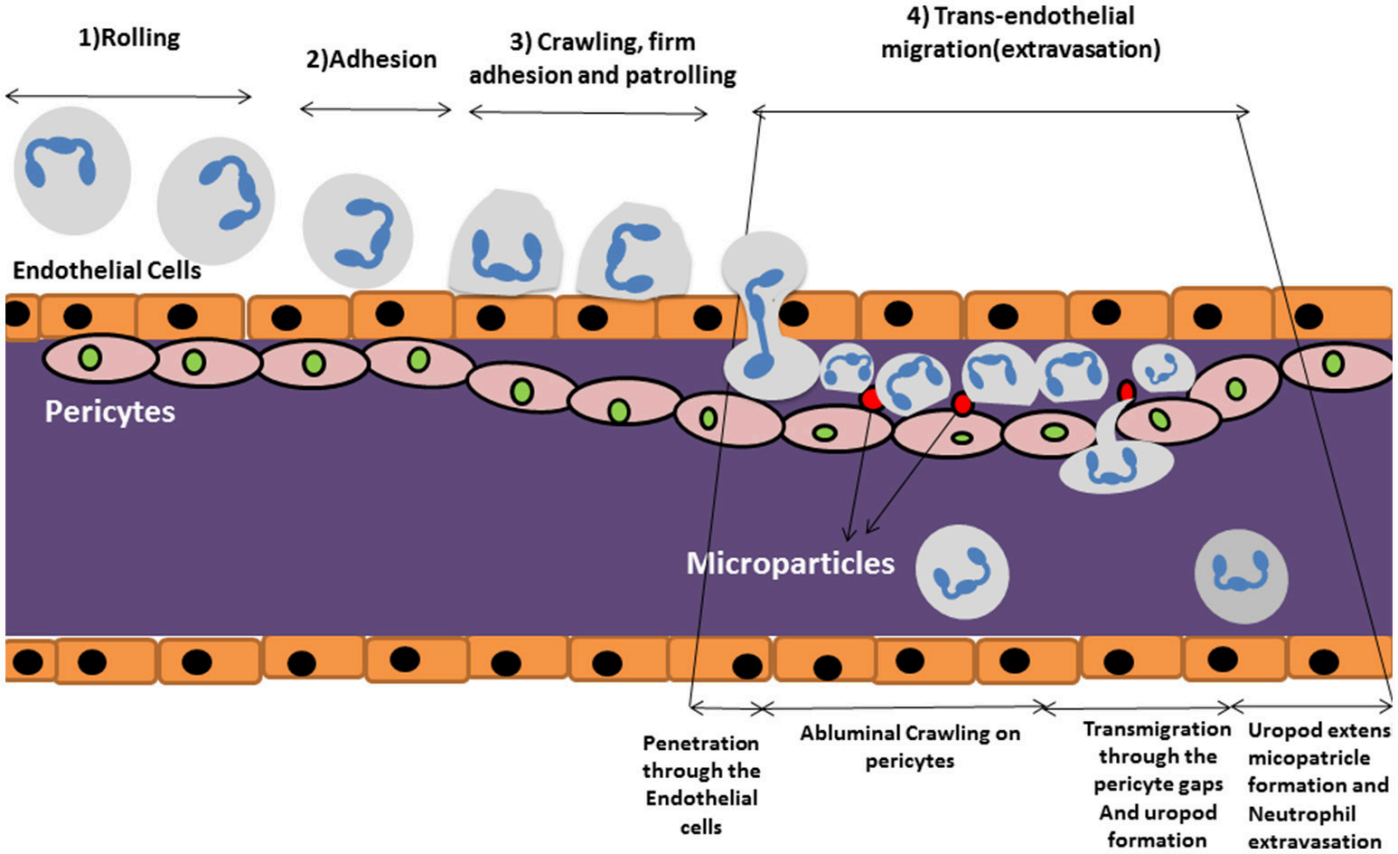
Schematic review of neutrophil extravasation cascade. The process of neutrophil migration begins with neutrophil “tethering to” the endothelium of blood vessels in steps (1) rolling, (2) adhesion, and (3) crawling, firm adhesion and patrolling. (4) Trans endothelial migration occurs after approaching the site of inflammation where they cross the blood vessel wall in an extravasation step in which neutrophils travel along the endothelial basement membrane until finding a small gap between pericytes. They start migrating through the space by forming a protruding uropod which allows neutrophils to access to the inflamed area. Microparticle formation occurs following uropod formation which is shown to have pivotal role in controlling vascular permeability.

Neutrophils quickly respond to inflammatory cues following infection or tissue damage and migrate to the inflamed/damaged area (26). Migration of neutrophils into the inflamed tissue, requires several steps that starts with adhesion to the endothelial surface followed by intravascular migration, extravasation and migration in the interstitium [(27); Figure 2].

Intravascular migration begins with neutrophil “tethering to” and “rolling on” the endothelium of blood vessels which is mediated by selectin molecules (21). Neutrophils then become activated by chemokines such as CXCL8 which trigger G-protein coupled receptors leading to a conformational change and activation of neutrophil integrin molecules such as VLA-4 (CD49D/CD29), MAC-1 (CD11b/CD18), and LFA-1 (CD11a/CD18) (21). This leads to an enhanced affinity for Ig-superfamily cell adhesion ligands (such as ICAM-1) expressed on the endothelium, which enables firm adherence of neutrophils to endothelial cells under flow conditions (17). Neutrophils then patrol the endothelial surface or migrate along a chemokine gradient to seek out the site of inflammation where they cross the endothelial layer in a processes generally referred to transendothelial migration or TEM (17).

Neutrophil extravasation through the endothelium occurs via either the paracellular or the transcellular route. The paracellular route involves leukocytes moving through endothelial cell junctions whilst the transcellular route involves neutrophil passage directly through the endothelial cell body (28). The paracellular route predominates in the majority of cases of neutrophil extravasation (29). Neutrophils travel along the endothelial basement membrane until they find a small gap between pericytes (27). Pericytes are contractile cells located at the abluminal site of microvessels and are responsible for controlling capillary permeability. Pericytes wrap around endothelial cells and cover 22–99% of the endothelial subcellular surface (30). These cells are also rich in pattern recognition receptors (PRRs) that enables them to sense inflammatory cues and act accordingly (31). The cells facilitate the extravasation process of the neutrophils to the tissues.

Once the neutrophil finds a gap between pericytes, it starts migrating through the space by forming a protruding lamellapodium (32). Elongation and passage of the lamellapodium through the pericyte/endothelial membrane is mediated by integrins such as MAC1, LFA-1, and VLA-3, respectively (26, 33). In the last step, extravasating neutrophils shed their CD18 integrins via vesicles from their extended tail or uropod at the subendothelial layer enabling retraction of their extended tail (26). This allows access to the inflamed area where the activated neutrophil will initiate engagement with micro-organisms and the clearance of cell debris.

### Extracellular matrix proteins and neutrophils activation

Neutrophils are strongly affected by their microenvironment including the presence of extracellular matrix (ECM). The effect of the ECM proteins such as collagen, laminin, fibronectin, and fibrinogen on inflammation has recently been explored and it is now evident that these proteins play a crucial role in providing signals that regulate different stages in neutrophil recruitment, transmigration and activation (1, 34–36).

The cytokine-induced respiratory burst in human neutrophils is dependent upon the interaction of ECM proteins with CD11/CD18 integrins (37). Subsequent studies have shown that the bovine neutrophil responses to IL-8 and platelet-activating factor (PAF) including intracellular calcium, actin polymerization, degranulation, adhesion, and oxidative burst changed dramatically after selective adhesion to different ECM proteins (38). Furthermore, the interaction between CD11b on neutrophils and the ECM protein fibrinogen, provided signals that enhanced the life span of neutrophils (39). ECM proteins also control neutrophil apoptosis indirectly by modulating tumor necrosis factor-alpha (TNF-α) expression within the local inflammatory milieu (39, 40). In contrast, the ECM proteolytic activity of neutrophils is critical for transmigration through the basement membrane (41).

Tissue damage can occur as a result of the neutrophils response to ECM protein signals in the inflammatory microenvironment (35). For example, in atherosclerosis the release of matrix modifying mediators such as neutrophil elastase (NE), myeloperoxidase (MPO), and defensins by activated neutrophils leads to the formation and development of atherosclerotic plaques (8, 42, 43). In the lung, the production and release of oxidants that results from the interaction of neutrophils with ECM proteins leads to injury and remodeling of the surrounding tissue matrix in COPD (44).

The interaction of ECM proteins with neutrophils also contributes to tumor metastasis. Chemokines produced by tumor cells activate microvascular endothelial cells inducing neutrophil adhesion and activation which is followed by the release of neutrophil oxidants and other matrix remodeling mediators. This results in a remodeling of the local microenviroment which facilitates the access of tumor cells to premetastatic sites (45). Greater research endeavors in this area may provide new therapeutic opportunities for the neutrophil-mediated inflammatory disorders.

### Neutrophil extracellular traps (NETosis)

Neutrophils as the first line of immune defense against pathogens and they utilize various mechanisms to eliminate microbes include phagocytosis, ROS production as well as the generation and release of microbicidal molecules following degranulation (6). More recently, another distinct antimicrobial activity of neutrophils has been described called NETosis (46). In 2004 it was reported that neutrophils could eject nuclear chromatin that was decorated with antimicrobial peptides and enzymes including defensins and cathelicidins as well as NE and MPO (47). This externalized chromatin structure or NETs was capable of killing or suppressing fungal and bacterial proliferation (46). There is much evidence to support the role of NETs in blocking microbial dissemination. In addition, mice deficient in MPO production and with an absence of NETs are susceptible to greater fungal dissemination (48). Furthermore, bacterial strains with mutations in a NET-degrading nuclease do not disseminate (48, 49).

The importance of NETs in immune defense is highlighted by their conservation across vertebrate species (50–53). Lipopolysaccharide (LPS) or protein kinase C activators can rapidly trigger (within minutes) the formation of NETs under extreme conditions such as severe sepsis (54). NET formation is categorized as an innate immune process and can be triggered by downstream intracellular mediators such as ROS which activate MPO and NE leading to chromatin decondensation (46).

NETs are associated with several pathological and infectious conditions and have the potential to prime other immune cells leading to sterile inflammation (46). There is also evidence for a role in autoimmune and inflammatory disorders (46). However, the beneficial or detrimental role of NETs in immune defense is controversial and several factors in the local infectious or sterile microenvironment can determine the impact of NETs to potentiate or suppress inflammation (48).

There is debate regarding the concept that NETosis be considered as a specific form of programmed cell death. Malachowa et al suggest that formation of NETosis is an incidental phenomenon rather than a result of programmed cell death (55). Indeed, NETosis may be considered as a beneficial effect of neutrophil suicide or cellular death. Leben and colleagues showed that phagocytosis of *S. aureus* pHrodo™ beads by human neutrophil granulocytes correlated with NETosis process and was dependent on NADPH oxidase activation in contrast to other pathways of cell death (56). However, there are contradictory results regarding the induction of NETosis according to the stimuli used (57).

It is unlikely that NETosis is the major mechanism by which neutrophils control infection as this cytolytic process involves the release of numerous DAMPs which would prolong and intensify the inflammatory response (55, 57). In recent years the concept of NET formation without neutrophil cell death, referred to as non-cytolytic or “vital NETosis,” has been introduced (57). This occurs via a ROS-independent mechanism (58) and is likely to be the normal manner by which NET factors are released.

## Neutrophil phenotypes and heterogeneity

The hematopoietic system consists of different subsets of myeloid and lymphoid cells with different phenotypes and functions (11). Beyond the differences in embryonic origin, this heterogeneity can be dictated by several elements including ontogenic and environmental factors (59). The presence of neutrophil heterogeneity was considered controversial for a long time because of their limited transcriptional activity, limited lifetime, and inability for reverse transmigration (RT) to peripheral blood after tissue homing. These did not match features of other heterogeneous cell populations (11). However, neutrophils acquire distinct phenotypes within their local microenvironment depending upon the physiological and pathological cues present (60).

Neutrophil heterogeneity is has been linked to survival time, function, density, NET-releasing capability and receptor expression profiles (61). Furthermore, distinct neutrophil subsets have been described based on the expression of cell surface markers (15). For example, CD66b/CD33 represents low-density neutrophils (LDNs) within the neutrophilic myeloid-derived suppressor cell (MDSC) population (62, 63) and CD49d positive neutrophils have been found in atopic individuals (64). The roles of these neutrophil subtypes in disease pathophysiology is unknown although some subtypes may be harmful whereas other neutrophil subsets may be beneficial at the sites of chronic inflammation. The next section will describe some receptor molecules and properties that characterize neutrophil subsets present in homeostasis and under pathologic conditions.

### Olfactomedin 4 (OLFM4) positive cells

The neutrophil granule protein olfactomedin 4 (OLFM4) defines two subtypes of neutrophils (OLFM4^+^ and OLFM4^−^) (11). These subtypes show no differences in apoptosis or antibacterial function *in vitro* and have an equal tendency for migration toward an inflamed area in response to inflammatory signals (65). OLFM4 gene knock-out mice exhibit reduced colonization of the gastric mucosa by *Helicobacter pylori (H. pylori)* which is associated with increased inflammatory cell infiltration, enhanced production of pro-inflammatory cytokines/chemokines such as IL-1β, IL-5, IL-12 p70, and MIP-1α and increased inflammatory response to *H. pylori* in gastric mucosa (66). It is speculated that OLFM4+ neutrophils localize to the NET of its parent cell during NETosis rather than increase NET formation *per se* and further studies are required to define the role of OLFM4+ neutrophils (39).

### CD177 (NB1) expressing neutrophils

CD177 is a 55 kDa glycosyl-phosphatidylinositol-anchored receptor that is expressed on human circulating neutrophils (67). CD177 has an important role in neutrophil transmigration through the endothelium as it has a high affinity for the adhesion molecule, platelet endothelial cell adhesion molecule-1 (PECAM-1) (68, 69). CD177 activation also modulates human neutrophil migration in a β2 integrin-dependent manner (70).

CD177 is also associated with the expression of the serine protease PR3 (68, 69). In human neutrophils, CD177 is co-expressed with PR3 on the surface of neutrophils and together these promote extravasation of circulating neutrophils (69). In severe bacterial infection the circulating levels of CD177+ neutrophils is augmented probably due to the elevated co-expression of PR3 which facilitates increased neutrophil tissue infiltration (71).

The circulating levels of CD177+ neutrophils are increased in patients with anti-neutrophil cytoplasmic antibodies (ANCA)-dependent vasculitis (70). Enrichment of these cells was associated with an increased risk of relapse (72). However, in ANCA-dependent vasculitis the expression of membrane bound PR3 is enhanced in primed CD177 negative neutrophils suggesting that anti-PR3-mediated neutrophil recruitment is independent of the role of CD177 (71). Interestingly, CD177+ neutrophils are the functionally activated neutrophil population in inflammatory bowel disease and negatively regulate disease (73). The role of CD177 in neutrophil migration and IBD currently seems to be more consensual than in the airways.

### CD63+ neutrophils

Single-cell analysis identified a subset of neutrophils in the airway of cystic fibrosis (CF) patients that appeared to have undergone functional reprogramming and acquired profound differences to circulating neutrophils including reduced intracellular glutathione and augmentation of lipid raft assembly (74). These neutrophils expressed CD63+, a marker of NE-rich granules, on their cell surface. In addition, expression of key phagocytosis receptors including CD16 and CD14 was enhanced (75). This was in contrast to the reduced levels of CD80 and of the prostaglandin receptor CD294 on these cells. This subset of neutrophils may represent an important future therapeutic target for airways disease (74).

### ICAM-1-expressing neutrophils

ICAM-1 (CD54) expressing neutrophils represent a population of tissue-experienced neutrophils that have migrated in a retrograde direction across endothelial cells and emerged again in the peripheral blood by reverse transmigration (75). This subpopulation of neutrophils are associated with chronic systemic inflammation (62). The function of these cells as well as their fate remain elusive.

### CD16^bright^CD62L^dim^ population and immune suppressive neutrophils

A CD16^bright^CD62L^dim^ population of neutrophils was first described by Pillay and colleagues as a unique circulating population of myeloid derived suppressor cells (MDSC) (74, 76). MDSCs were originally identified in a murine model of cancer as a population of heterogeneous immature myeloid cells that suppress immune responses (77–79). Gabrilovic et al. in 2007 subsequently coined the term MDSCs to emphasis their heterogeneity (80).

The CD16^bright^CD62L^dim^ population of neutrophils can mimic MDSCs and exhibit a suppressive function and suppress T-cell proliferation *in vitro* while remaining remarkably poor at eliminating bacteria such as *Staphylococcus aureus (S. aureus)* (81). This suppressive immunophenotype of mature neutrophils is also detected in peripheral blood samples of cancer patients suggesting their involvement in antitumor immunity (82). The CD16^bright^CD62L^dim^ population will be discussed in greater detail in section Neutrophil Phenotypes After Trauma.

### Pro-angiogenic neutrophils

A subpopulation of CD11b+/Gr-1+ neutrophils in the mouse was first described in 2012 as being recruited to transplantation sites and having the ability to promote re-vascularization of the transplanted tissue (83). This population of neutrophils at least in the mouse express high levels of CXCR4 (CXCR4^hi^). In an *in vivo* model they can deliver large amounts of MMP-9 to transplanted islets of Langerhans. This in turn, induced VEGF-dependent angiogenesis at the site of recruitment (84). These cells have yet to be found in humans *in vivo*.

### Low density neutrophils (LDNs)

Low-density neutrophils (LDNs) comprise a population of neutrophils with a low buoyant density that are typically found in the mononuclear fraction upon density centrifugation. These cells include cells both segmented and banded nuclei as well as myelocyte-like progenitor cells (85). LDNs were first reported in systemic lupus erythematosus (SLE), rheumatoid arthritis and rheumatic fever (86). They are now also recognized as being elevated in cancer and are in some studies associated with tumor progression (87). The cells are responsible for the down-regulation of T-cell function via an arginase dependent mechanism such as found during the induction of materno-fetal tolerance (87).

In sepsis, LDNs are present and play a pivotal role in sepsis-induced immune suppression. In patients with sepsis, granulocyte-like MDSC which include a low density granulocyte (LDG) population, help drive T-cell dysfunction by the production of arginase 1 that enables the subsequent development of nosocomial infections (88).

LDNs from patients with SLE have a higher propensity to form NETs in a process referred to as NETosis (89) and to release pre-formed NETs. NETs can present auto-antigens to the immune system suggesting that LDNs, from SLE patients at least, can promote chronic inflammation leading to autoimmunity (89, 90). High CD66b+ LDN counts were also reported in the peritoneal cavity of patients with gastric cancer following abdominal surgery (91). These CD66b+ LDNs have the ability to form NETs and to selectively capture disseminated tumor cells (91).

The function of LDNs is dependent on the local microenvironment and the associated pathology (92). For example, in cancer LDNs have an immunosuppressive activity (93) whilst they generally possess a pro-inflammatory phenotype in autoimmunity disease (94, 95). In SLE, activated LDNs produce high levels of type 1 interferons (INFs) (95). LDNs with an activated phenotype have also been reported during leishmania infection (94, 96). However; LDNs play a major immunosuppressive role in sepsis which is associated with higher incidence of nosocomial infections (88). The characterization of these cells *in vivo* is difficult due to the lack of specific cell surface or molecular markers (97).

### Tumor associated neutrophils (TANs)

Mature neutrophils that leave the bone marrow and are released into the circulation may migrate into tumors where they can be found as tumor associated neutrophils (TANs) (98). After infiltration to tumor sites, these neutrophils undergo profound phenotypic changes compared to their circulating counterparts (99).

In murine models of cancer, TANs showed two distinct populations referred to as N1 and N2 with pro- and anti-tumoral roles, respectively (100). Transcriptomic analysis showed that N1 and N2 neutrophils have distinct gene expression profiles and functional properties which are likely induced by the local tumor microenvironment (101). The role(s) of TANs in the tumor microenvironment in man remains unclear although TANs isolated from human lung tumors possess an activated phenotype (CD62^Low^CD54^hi^) with increased pro-inflammatory cytokine production. These TANs also induce T-cell proliferation and the production of IFN-γ (98).

In contrast, in colorectal cancer TANs produce arginase I and ROS thereby inhibiting proliferation and IFN-γ production by T-cells (102). These data suggest that these cells exhibit LDN-like properties (102). The numbers and functions of TANs are regulated by chemotherapy in human colorectal cancer which supports the hypothesis that they have a role in cancer progression (103). However, the degree to which TAN subsets are present in human cancers or whether different human cancers include different TAN subsets is unclear.

Despite the current consensus around the existence of neutrophil heterogeneity, there are still some challenges to be met. It is possible that the different observed properties of the neutrophil populations merely reflect the response to the local environment and differences result from their innate plasticity (93). Further studies are required to link the various phenotypic characteristics with cellular/tissue functions.

## The importance of neutrophils in inflammatory complications found after trauma

Trauma is the main case of mortality worldwide in people under the age of 50 (12). In 5% of cases, patients suffer from severe trauma. This clinical condition can ultimately lead to multiple organ dysfunction syndrome (MODS) in which the functionality of some organs such as liver, lung or kidney is markedly reduced (104). The main cause of post-traumatic complications is due to hyperactivation of the immune response (105). For example, a localized inflammatory reaction after trauma can be provoked by alarm signals (alarmins and other damage associated molecular patterns/DAMPS) which are secreted by healthy, damaged or necrotic cells (106).

Neutrophils are important effector cells in managing and regaining tissue homeostasis and in the maintenance of immune surveillance. Activation of neutrophils after trauma by alarm signals evokes the development of a local inflammatory response. If this local inflammatory response becomes excessive this may lead to a SIRS and MODS.

**Figure 3:**
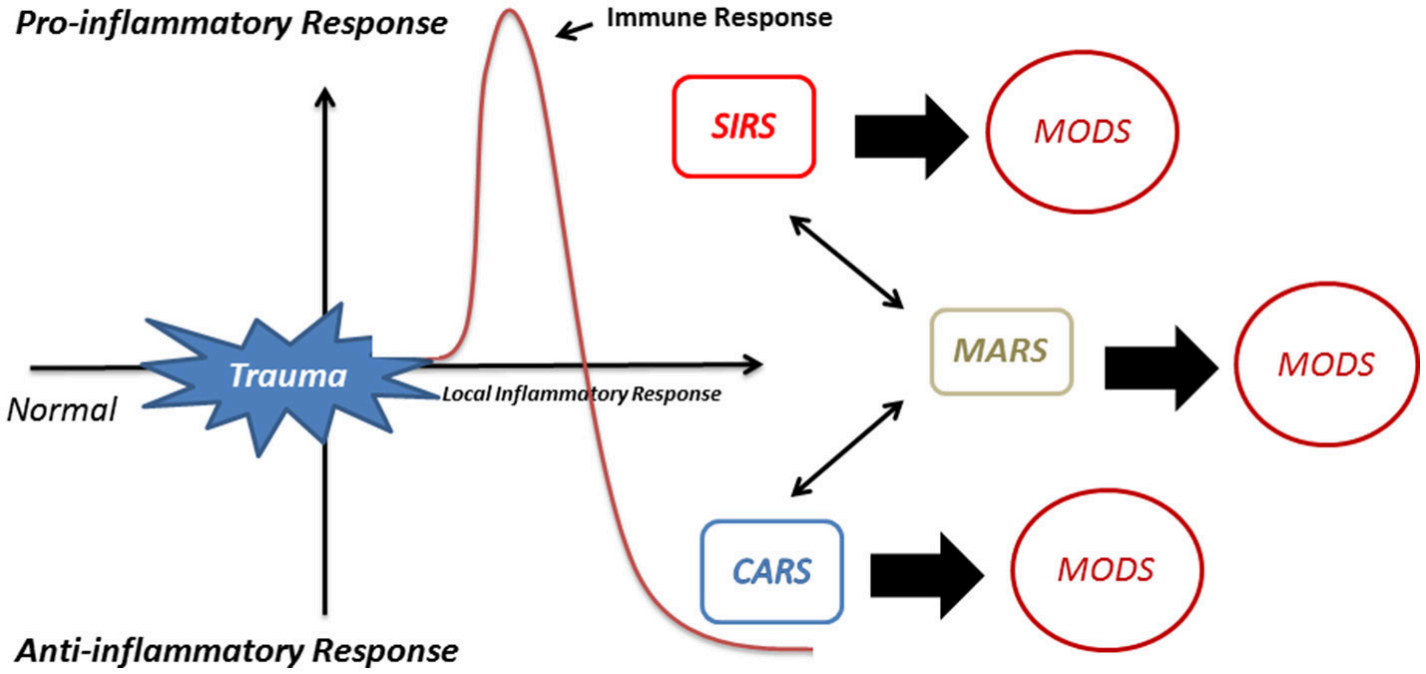
Activation of immune response after trauma. Activation of neutrophils after trauma evokes the development of a local inflammatory response. If this local inflammatory response becomes excessive this may lead to a systemic inflammatory response (SIRS) and multiple organ dysfunction syndrome (MODS). To restore the equilibrium to a favorable state, a compensatory anti-inflammatory response (CARS) may occur or, alternatively, the pro-inflammatory and anti-inflammatory responses may counteract leading to a mixed antagonist response (MARS).

MODS has a mortality rate of up to 50–80% (106). To control this disproportionate pro-inflammatory reaction and to restore the equilibrium, a compensatory anti-inflammatory response (CARS) may occur (105, 107). Alternatively, the pro-inflammatory and anti-inflammatory responses may counteract each other leading to a mixed antagonist response (MARS) (108, 109). Both CARS and MARS tune down the disproportionate immune activation (110) making the patient extremely susceptible to infection by microorganisms. This results in serious complications such as sepsis, septic shock and organ failure [(105); Figure 3].

Neutrophils play an important role in the pathophysiology of the deregulated immune response found in patients with trauma (111). Tissue damage leads to neutrophil activation and the production of ROS due to various triggers such as hypoxia and reperfusion injury in damaged tissue and the release of neutrophil chemoattractants and activators (112). The presence of neutrophil priming agents such as granulocyte-macrophage colony-stimulating factor (GM-CSF) or TNF-α in the peripheral blood, enhances neutrophil chemotaxis, extravasation and oxidative burst production (113). The spontaneous production of ROS by neutrophils is elevated in trauma patients and the uncontrolled ROS production by accumulated neutrophils in the vascular bed increases vascular permeability promoting organ failure (112).

Selectins and integrins mediate neutrophil transmigration toward the inflamed and/or damaged tissue. Neutrophils release L-selectin during migration and serum levels of L-selectin (sL-selectin) are associated with the degree of neutrophil activation. Maximum sL-selectin levels were observed 6 h after trauma (113, 114). The destructive effects of neutrophils within tissue is limited by neutrophil apoptosis. However, this process is delayed after trauma (115). Delayed neutrophil apoptosis leads to the accumulation of neutrophils, increased release of their cytotoxic products and the promotion of local tissue damage (105, 116).

Although neutrophils are activated during SIRS post-trauma, their responsiveness to the innate stimulus fMLP decreases. This is illustrated by the decreased expression of active FcγRII (CD32) induced by fMLP on neutrophils in poly trauma patients. Consequently, the low functionality of this most important Fcγ receptor on neutrophils probably involves the decrease of antibacterial function during CARS over the following days (117, 118). This may be due to the production of CD16^low^ immature neutrophils (118). The recruitment of immature band forms of neutrophils from the bone marrow into the circulation is typically found in sepsis and SIRS (119). It is yet to be determined whether these young cells are dysfunctional or whether these cells are fully functional as is seen after LPS challenge (81). On the other hand, these immature neutrophils show lower expression of antibacterial receptors such as CD14 and MD-2 and have a reduced transmigration ability (118).

The endocrine system also modifies neutrophil changes after severe injury. Both trauma-induced cortisol and epinephrine strongly increase neutrophil release into circulating blood (120). Cortisol is also thought to extend the half-life of circulating neutrophils (120). Together with the reduced chemotaxis found after cortisol and epinephrine infusion these combined effects could account for increased susceptibility to infection observed after major trauma (120).

### Neutrophil phenotypes after trauma

Acute inflammation is accompanied by the recruitment of neutrophils with different phenotypes into the circulation that are not present during homeostasis (12, 121). These “inflammatory” neutrophils have distinct characteristics such as enhanced expression of CD124 (IL-4Rα), CD15 (3- fucosyl-N-acetyl-lactosamine), and arginase in addition to a lower buoyant density and immunomodulatory properties (3). In 2012, Pillay et al. observed neutrophil subtypes in the circulation during experimental acute systemic inflammation evoked by systemic administration of 2 ng/kg LPS in human healthy volunteers (3). Based on the expression level of CD16 (FcγRIII) and CD62L (L-selectin), three different subsets of “inflammatory” neutrophils were observed: neutrophils with a conventional segmented nucleus (CD16^bright^/CD62L^bright^), neutrophils with a banded nucleus (CD16^dim^/CD62L^bright^), and CD62Ldim neutrophils (CD16^bright^/CD62L^dim^) with a higher number of nuclear lobes (hyper-segmented).

Banded neutrophils observed in acute inflammation are fully functional and are superior in killing *S. aureus* (81). In contrast, CD62L^dim^ neutrophils were enriched by proteins involved in immune regulation (122) as these cells have immunosuppressive properties and inhibit T-cell proliferation (123). CD16L^dim^ neutrophils also showed lower cell adhesion capacity and an extremely low capacity to contain bacteria in comparison to the two other subtypes (124). Chemotaxis toward end target chemo-attractants was also decreased in this group which might result in reduced endothelial binding and extravasation to inflammatory sites (124). The origin of CD62L^dim^ cells after trauma in humans is not clear but these cells do not represent aged cells (3).

Although it is commonly believed that increased nuclear segmentation occurs with increasing cellular age this is not really supported by experimental data. For example, in humans the hyper-segmented neutrophils seen in pernicious anemia result from defects in the DNA replication machinery. These hyper-segmented neutrophils appear in the circulation simultaneously with normal neutrophils (124). In addition, a study applying proteome profiling and *in vivo* kinetics of neutrophils following LPS challenge showed that hyper-segmented neutrophils have a similar age as normal segmented cells and take the same time to reach maturity and, as such, cannot be considered as aged cells (125). Furthermore, these hyper-segmented CD62L^dim^ cells do not seem to originate from mature neutrophils but might be produced by a separate pathway compared to banded and normal segmented neutrophils in response to inflammation (123). These cells enter the bloodstream only during inflammation as a distinct neutrophil subset. It is, therefore, tempting to speculate that these cells have a specific goal of fine-tuning the acute immune response (96).

### The role of neutrophils in immune dysfunction after trauma and inflammation

Neutrophils are involved in the deregulation of immune responses during trauma by several mechanisms including cleavage of essential cell surface receptors, modulation of the function of immune receptors, control of peripheral blood neutrophil numbers, and modulation of the adaptive immune response.

#### Cleavage of essential cell surface receptors

Neutrophil-derived serine proteases released by degranulation can mediate proteolytic cleavage of receptors on immune cells (126). During acute inflammation NE can cleave and downregulate the expression of the IL-8 receptors CXCR1 and 2 (127, 128). This, in turn, decreases the responsiveness to IL-8 and enhances the risk of pneumonia in trauma patients (129). Complement receptors may also be targeted by neutrophil proteases. Decreased levels of CR1/CD35 (130) and of C5aR/CD88 have been reported during inflammation which might result in a failure of neutrophil engagement with micro-organisms (131). Cleavage of CD14 on the surface of monocytes can also be affected by NE which can lead to impaired TLR4-mediated recognition of lipopolysaccharide by monocytes (132). On the other hand, NE can also modulate adaptive immune responses by enhancing the shedding of the IL-2 and IL-6 receptors on T lymphocytes (133).

#### Desensitization of immune receptors

After severe injury and trauma, suppression of immune function may occur due to the desensitization of immune receptors on neutrophils (133). Trauma/severe injury leads to the release of endogenous danger signals (danger-associated molecular patterns; DAMPs) from necrotic tissue cells that can bind to PRRs on neutrophils (134). DAMPs, which alert the immune system in response to stress, resemble pathogen-associated molecular patterns (PAMPs) and can bind to their receptors on neutrophils (135). Finally, DAMPs released during trauma can induce both homologous and heterologous desensitization of immune receptors via internalization of PPRs which limit subsequent responses to microbial signals (136).

#### Regulation of neutrophil numbers

The number of neutrophils in the circulating blood is regulated by the CXCL12/CXCR4 axis in the mouse (24). Expression of the chemokine CXCL12 by bone marrow stromal cells provide a signal for neutrophils expressing the CXCL12 receptor CXCR4 to remain in the bone marrow (22). Conversely, attenuation of CXCR4 signaling leads to the release of neutrophils into the circulation. Disruption of the CXCR4/CXCL12 balance by inflammatory stimuli can increase neutrophil release into peripheral blood (24) or can lead to leukostasis in the bone marrow such as found in WHIM syndrome.

In human experimental endotoxemia, peripheral neutrophils exhibit a functional heterogeneity and different degrees of priming (122). Similar variations in neutrophil phenotypes are seen in the peripheral blood of patients during severe inflammation (137). A large number of immature or banded cells, suppressive neutrophils, myeloid-derived suppressor cells, and neutrophil progenitor cells can be detected (119). The presence of immature or banded neutrophils may be either a compensatory response due to depletion of mature neutrophils in bone marrow or it may be induced by the bacterial stimulus itself (123).

#### Modulation of the adaptive immune response

Neutrophils play the major role in the paralysis of the immune system during CARS that may occur because of systemic inflammation (138). Immune paralysis is an immunosuppressed state in which immune responses are unable to recover despite the clearance of pathogens. This leads to a failure in the ability to control the primary infection and increased susceptibility to secondary infections. Immune paralysis is the main cause of death in most sepsis patients (139).

Apart from their roles in innate immunity and direct anti-microbial defense, neutrophils can also modulate adaptive immune cells in severe inflammation (119). Neutrophils can modulate T-cell responses via different mechanisms. A T-cell found in an inflammatory microenvironment may be affected by neutrophil-derived chemokines and cytokines or by their released granular contents (140). NE can directly cleave receptors on the T-cell surface such as those for IL-2 and IL-6 (133). NE can also reduce the level of co-stimulatory molecules on dendritic cells and subsequently decrease T-cell maturation (141).

Macrophages shift toward an anti-inflammatory phenotype after phagocytosis of apoptotic neutrophils (142). MDSCs are a heterogonous population consisting of myeloid progenitors, immature neutrophils, macrophages and dendritic cells that expand during a wide range of pathological conditions such as cancer and inflammation (143). These cells are potent suppressors of various T-cell functions in mouse models. These cells can produce arginase-1 and thereby deplete arginine from the local microenvironment (144). Arginine is an essential amino acid and its depletion leads to cell cycle arrest of T-cells in the G0–G1 phase (145). A subset of neutrophils have been identified in the peripheral blood of patients with septic shock that can secrete arginase-1 and function as MDSCs (146).

In man, a systematic LPS challenge induced the release of a subtype of mature CD62L^dim^ neutrophils with a hyper-segmented nuclear morphology into the circulation (123). These cells suppressed T-cell function via a Mac-1/(αMβ2)-dependent (CD11b/CD18-dependent) mechanism and delivery of hydrogen peroxide into the immunological synapse (123). A similar subset has been found after systemic treatment with G-CSF (147). However, the latter cells employ arginase rather than oxidants to suppress T-cell function *in vitro*.

Proteome profiling of L-selectin/CD62L low neutrophils showed that this subtype is enriched in proteins involved in immune regulation and exhibited a marked decrease in ribosomal proteins compared to immature banded neutrophils (3). This implied that the L-selectin low cells lost a significant part of their protein translational machinery (3).

**Figure 4:**
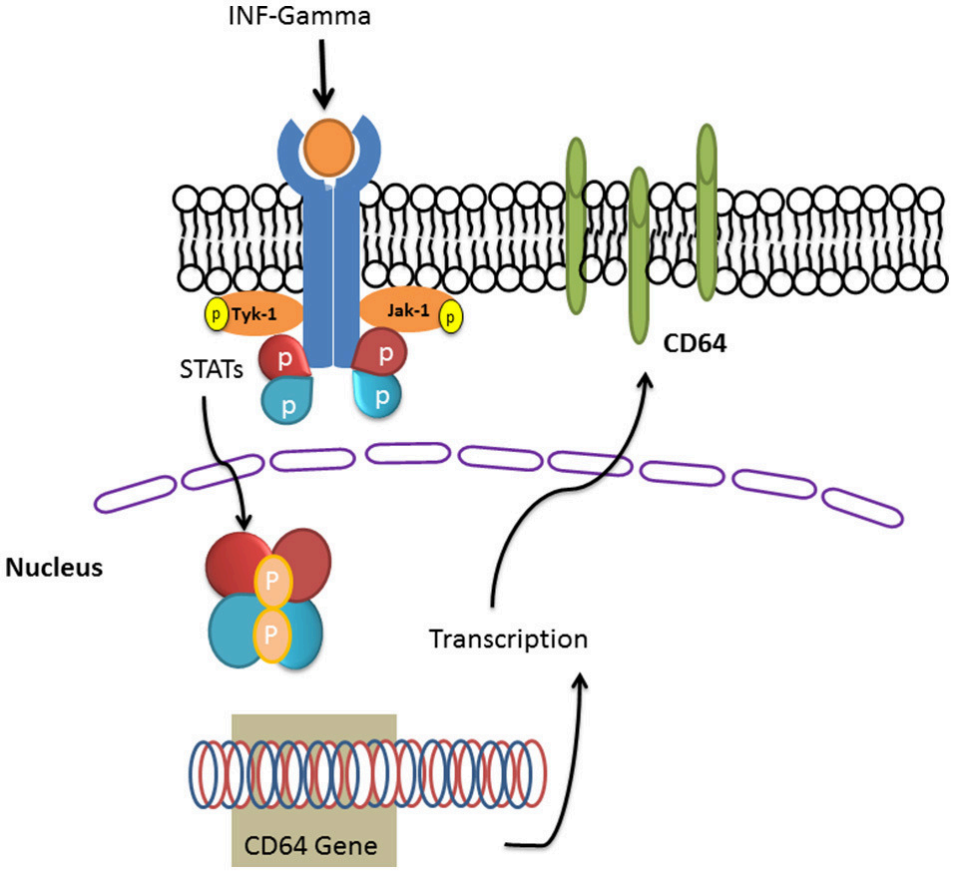
INF-gamma induced the expression of Fc gamma receptor (CD64). IFN-γ induces its receptor and the downstream signaling pathways including JAKs and the STAT family of transcription factors. STAT dimers enhance the transcription of CD64 gene and the translated CD64 will be targeted in to the plasma membrane bilayer lipid.

INF-γ induces the expression PD-L1 by neutrophils which enables them to suppress lymphocyte proliferation and induce lymphocyte apoptosis (148). Blocking this PD-1/PD-L1 axis in a murine model of sepsis reversed immune dysfunction and improved survival (149). INF-γ also induced the expression of Fc gamma receptor (CD64) by induction of transcription factor STAT1 [(150); Figure 4].

Neutrophil subgroups also play a role in cancer immunity. CD16^high^CD62L^dim^ cells are more common in patients with head and neck squamous cell carcinoma (HNSCC). These cells produce NETs, displayed an activated phenotype and, in comparison to other subtypes, were more prone to migrate to tumor sites and perform anti-tumor immune functions including inhibition of proliferation and the induction of apoptosis in cancer cells. An increase in circulating CD16^high^CD62L^dim^ neutrophils was associated with increased NET formation and increased survival in HNSCC patients (151).

## The application of neutrophils as biomarkers in infectious diseases

The neutrophil Fcγ-receptor I (FcγRI, CD64) has long been considered as a biomarker for infectious disease. It is a high affinity receptor that binds to the Fc part of the IgG heavy chain (152). CD64 is normally expressed at a very low level on the surface of resting neutrophils in healthy individuals (153) but its expression is markedly elevated within a few hours of bacterial infections (154). The expression can be elevated >10-fold which allows differentiation between resting and activated neutrophils (155, 156). This property of CD64 has been utilized as a diagnostic marker of infection (156) particularly in sepsis. The expression of this marker is very stable after blood collection and it requires only a small volume for assessment (156) making this a very attractive marker for infection monitoring.

The level of CD64 is moderately elevated in preterm newborn infants before changing to normal levels in the first month of life (157). However, a meta-analysis of the diagnostic accuracy of neutrophil CD64 in neonatal sepsis suggests that this alone cannot be used as a satisfactory marker for neonatal sepsis due to its relatively low sensitivity and specificity (158). However, neutrophil CD64 levels are a very sensitive diagnostic marker for early-onset clinical infection and pneumonia in newborns and can guide antibiotic therapy (159). In addition, circulating neutrophils in Erythema nodosum leprosum (ENL) patients expressed CD64 on their cell surface and this expression was correlated with disease severity (160).

Neutrophil gelatinase-associated lipocalin (NGAL) is another neutrophilic marker important for the early diagnosis of acute kidney injury (161). NGAL is also used as a biomarker for inflammation in cardiovascular disease including atherosclerosis, heart failure as well as acute myocardial infarction (161).

A novel neutrophil derived inflammatory biomarker in CF patients has been recently introduced (162). This is a protein complex containing alpha-1 antitrypsin and CD16b (AAT:CD16b) that is released into the bloodstream from membranes of pro-inflammatory primed neutrophils. The plasma level of AAT:CD16b complex correlates with inflammatory status in CF patients and has been proposed as a biomarker for the diagnosis of CF exacerbations (162). In ulcerative colitis (UC), the presence of human neutrophil lipocalin (HNL), and MPO in colorectal perfusion fluids indicates intestinal neutrophil activation in UC (163).

## Conclusion

Neutrophils are main players in the context of inflammatory complications during and after infections and tissue injury. The neutrophil compartment is heterogeneous and neutrophils with distinct properties have been identified. These cells exhibit a high plasticity and easily adapt to changes in microenvironment. Newly identified human neutrophil subsets can suppress T-cell activation and proliferation and their presence may provide a novel therapeutic and/or diagnostic avenue in chronic and acute infection as well as in cancers. In contrast, neutrophils also play the central role in the immune paralysis after severe inflammation and have a detrimental role in organ failure in post-injury events.

The paradox regarding the role of neutrophils in health and disease dictates that therapy should be targeted to the correct phenotypes to prevent off-target effects. It is now of paramount importance to identity the site of origin of different neutrophil phenotypes present in severe inflammation. The potential of neutrophil cell surface markers or their products to be used in diagnosis or in therapy has great potential but requires further study. In addition, the mechanism(s) by which neutrophils drive immune paralysis and subsequent tissue damage post-trauma are an important therapeutic target as there is great unmet medical need to control neutrophil activation in most inflammatory diseases.

## Author contributions

EM and SA wrote the original draft of manuscript. SM helped with editing, literature collating and referencing. LK and IA revised and edited the manuscript.

## Conflict of interest statement

The authors declare that the research was conducted in the absence of any commercial or financial relationships that could be construed as a potential conflict of interest.
